# Atypical Influenza A(H3N2) Activity Patterns in Germany, 2021–2023, and Characterization of Newly Emerged Virus Clades

**DOI:** 10.1002/jmv.70530

**Published:** 2025-08-07

**Authors:** Susanne Duwe, Djin‐Ye Oh, Marianne Wedde, Daniela Börnigen, Ralf Ignatius, Max von Kleist, Janine Reiche, Barbara Biere, Thorsten Wolff, Ralf Dürrwald

**Affiliations:** ^1^ Robert Koch‐Institute, Department 1: Infectious Diseases Unit 17: Influenza and Other Respiratory Viruses Berlin Germany; ^2^ Robert Koch‐Institute Project Group 5: Systems Medicine of Infectious Diseases Berlin Germany; ^3^ Medizinisches Versorgungszentrum Labor 28 GmbH Berlin Germany; ^4^ Department of Mathematics and Computer Science Free University Berlin Germany

**Keywords:** antigenic analysis, influenza, surveillance, inhibition test, phylogenetic analysis, resistance, respiratory infection, vaccine, virological analysis

## Abstract

The first waves of the COVID‐19 pandemic were accompanied by an unprecedented decrease of influenza activity which persisted throughout the 2020/21 and 2021/22 winter seasons. Here, we report on the unusual influenza circulation patterns that followed in the year 2022, which was dominated throughout by A(H3N2) influenza viruses. After a delayed spring wave in 2022, A(H3N2) influenza viruses circulated at low levels throughout the summer and rose to a prominent, prematurely‐timed fall/winter wave peaking in December, with highest positivity rates observed in 10–12‐years old children. This winter wave ended abruptly with the national school holidays, when positivity rates decreased sharply not only in children but also in other age groups. Genetic analysis of influenza virus hemagglutinin (HA) showed cocirculation of 10 A(H3N2) clades, of which three (2a.1b, 2a.3a.1, and 2b) became dominant in late 2022. All A(H3N2) viruses, including those assigned to the new clades, displayed high titers in HA inhibition tests with postinfection ferret antiserum raised against the A(H3N2) vaccine strains A/Cambodia/e0826360/2020 and A/Darwin/9/2021. All viruses were susceptible to neuraminidase inhibitors and the polymerase inhibitor baloxavir marboxil, but carried the M2‐S31N substitution conferring adamantane resistance. Our findings shed light on disturbed seasonality of A(H3N2) circulation in the post‐COVID‐19 era.

## 1 Introduction

Influenza is a highly contagious respiratory virus that is easily transmitted via aerosolized droplets. Influenza virus infections are associated with substantial morbidity and mortality, causing an annual burden of approximately three to five million cases of severe illness, and 300.000 to 500.000 deaths worldwide [1, 2]. Historically, influenza virus circulation follows pronounced seasonal patterns, which depend on geographical location and climatic conditions [3]. In temperate regions of the Northern and Southern hemisphere, influenza epidemics usually occur in seasonal winter waves [2, 4, 5]. In Germany, the seasonal influenza wave typically starts in late December and reaches peak activity in February [6, 7]. These typical patterns were disrupted during the COVID‐19 pandemic, when nonpharmaceutical interventions (NPIs) applied to curb the spread of SARS‐CoV‐2 led to a substantial decrease of global influenza activity throughout the 2020/21 season [6, 8]. During 2022, most if not all NPIs were lifted; accordingly, a substantial rise of respiratory virus activity was observed worldwide. The first indication of this global resurgence was the exceptionally intense influenza season in Australia during the Southern hemisphere winter in May/June 2022 [9]. In August 2022, Italy reported the out‐of‐season circulation of influenza viruses and an atypically timed influenza outbreak that occurred in the Southern Italian region of Apulia [3]. Slightly elevated influenza activity was also observed in other countries of the Northern hemisphere and influenza viruses continued to circulate at low levels throughout the 2022 summer months [8].

In Germany, similar toother parts of Europe, influenza virus activity was practically nonexistent during the 2020/21 season amid extensive pandemic restrictions. Although a gradual relaxation of these restrictions began during the 2021/22 season as the COVID‐19 vaccination campaign proceeded, multiple public health measures were continued throughout the 2021/22 winter, until the vast majority of anti‐pandemic measures were aborted in March 2022 [10, 11]. Subsequently, there was a notable increase in influenza activity. The objective of this study was to comprehensively characterize the circulation patterns and virological properties of the A(H3N2) viruses driving the influenza resurgence in Germany after antipandemic restrictions were eased.

## 2 Material and Methods

***Ethics approval and patient consent.*** Written approval for the German national surveillance of influenza and other respiratory viruses was obtained from the Charité‐Universitätsmedizin Berlin Ethical Board (reference EA2/126/11). All analyses were based on pseudonymized data. Written informed consent was obtained from all sentinel patients.

***Surveillance.*** of respiratory viruses and virological characterization of influenza viruses was conducted using clinical specimens from ambulatory patients participating in Germany's national sentinel system for monitoring acute respiratory infections (ARI) [6, 12, 13, 14]. The reporting period for each influenza season starts in Week 40 and extends through Week 39 of the following year; accordingly, clinical specimens were sampled between Weeks 40/2021 and 39/2023. Samples were analyzed for influenza and other respiratory viruses using multiplex RT‐PCRs. Selected influenza viruses underwent genetic, antigenic and resistance characterization.

***qPCR.*** Influenza A viruses were detected and subtyped using in house multiplex oligonucleotide systems as previously described [6, 15, 16].

***Virus isolation in MDCK‐SIAT cells.*** Influenza viruses were isolated by inoculating MDCK‐SIAT cell monolayers (ECACC, United Kingdom) as described elsewhere [17, 18, 19].

***Antigenic characterization.*** Antigenic characterization of influenza viruses was performed by hemagglutination‐inhibition assays (HAI), using specific antisera generated in ferrets [20]. Antisera were raised against the Northern Hemisphere A(H3N2) vaccine strains of the respective season, that is, A/Cambodia/e0826360/2020 in 2021/22 and A/Darwin/9/2021 in 2022/23.

***Serological investigations.*** Residual serum specimens sampled in the catchment area of Berlin in August 2024 (100 per age group: 0–4, 5–15, 16–34, 34–60, 61–80, > 80 years) were investigated by HAI using A/Thailand/8/2022 A(H3N2) virus.

***Sequencing.*** Influenza A(H3N2)‐positive samples underwent RNA extraction, virus‐specific multi‐segment RT‐PCR amplification and whole generation sequencing (WGS). Libraries were generated with Nextera XT (Illumina) and sequencing was performed on a Nextseq. 2000 system (Illumina) with 150 bp paired‐end reads [12, 21].

***Phylogenetic analysis.*** Phylogenetic analysis of the hemagglutinin (HA) gene of influenza A(H3N2) viruses was performed using Mega11 (NJ, K2, bootstrap test with 1000 replicates, partial deletion: site coverage cutoff 5%). All consensus sequences were submitted to GISAID (Table S1).

***Determining antiviral susceptibility.*** The susceptibility of influenza viruses to neuraminidase (NA) inhibitors was measured in a fluorometric NA inhibition assay using 2′‐(4‐methylumbelliferyl)‐α‐d‐N‐acetylneuraminic acid (Munana; Biosynth AG, Switzerland) as substrate; 50% inhibitory concentrations (IC_50_) were assessed against reference IC_50_ values according to WHO criteria [18, 22].

Consensus sequences of viral genomes were analyzed for molecular markers of resistance against oseltamivir, zanamivir, amantadine, rimantadine, and baloxavir marboxil as previously described [13].

## 3 Results

### 3.1 A(H3N2) Influenza Virus Circulation in Germany, Seasons 2021/22 and 2022/23

**Figure 1:**
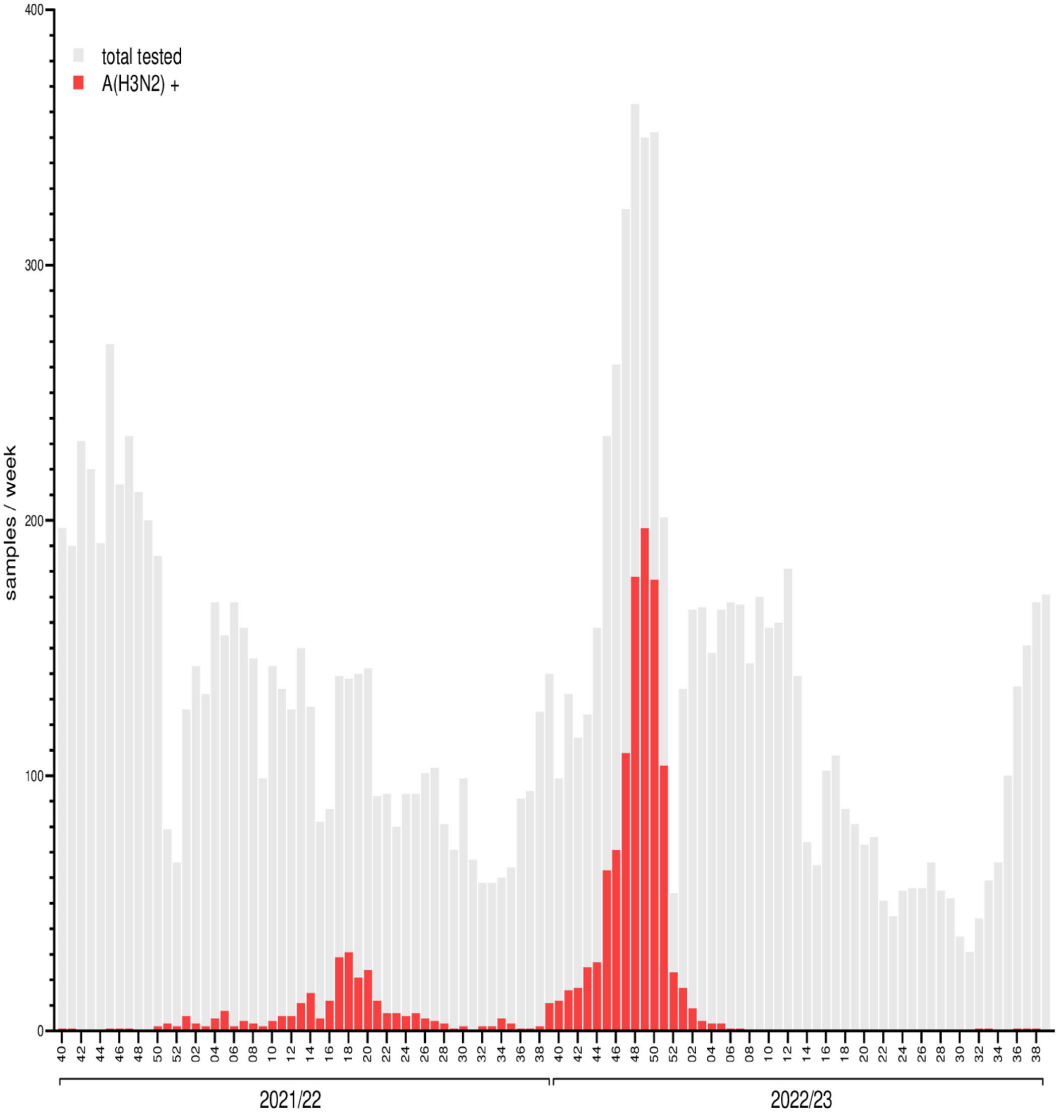
Influenza A(H3N2) detections in the German ARI sentinel, 2021/22–2022/23. Absolute numbers of sentinel samples testing positive for influenza A(H3N2) (red bars) in comparison to total sentinel samples tested (shadow bars) per week. The reporting period for each influenza season starts in Week 40 and extends through Week 39 of the following year; the *X*‐axis is labeled accordingly.

In the context of the nationwide lab‐based respiratory virus surveillance, a total 13 746 specimens from ambulatory patients with ARI symptoms, presenting in sentinel clinics between Week 40, 2021 and Week 39, 2023, underwent diagnostic PCR analysis at the National Influenza Center. Of these, 1354 samples, that is, the vast majority (91%) of a total 1483 influenza A‐positive samples, tested positive for influenza A(H3N2). Analysis of the temporal distribution of influenza A(H3N2) positive samples revealed that most (46 of 52) Weeks of the 2021/22 season featured at least one influenza A(H3N2) positive sentinel sample, indicating persistent A(H3N2) circulation throughout 2021/22 (Figure 1). Detections were somewhat sporadic until December 2021, when a slight uptick in the number of positive samples was noted. From Week 10 (March 2022) on, detections rose gradually, with the highest numbers of A(H3N2) positives noted in Weeks 17 to 20 (May 2022); afterwards, positive sample numbers declined. Notably, A(H3N2) detections continued throughout the summer with an uptick at the end of the season. Coinciding with the start of the 2022/23 influenza season in Week 40/2022, the number of influenza A(H3N2) positive sentinel samples increased sharply and robustly, until a peak was reached in Week 49/2022. This prominent A(H3N2) influenza wave ended abruptly at the end of the year. A(H3N2) detections in the sentinel continued at low, declining numbers until Week 7/2023 (February 2023). Subsequently, there was no indication of A(H3N2) activity until sporadic detections resumed in Week 32/2023 (August, 2023) (Figure 1).

### 3.2 Temporal Patterns of Influenza A(H3N2) Circulation Over 14 Seasons (2009/10–2022/23)

To evaluate whether influenza A(H3N2) circulation in 2021/22 and 2022/23 followed typical seasonal patterns, national virological surveillance data spanning a time period of 14 seasons from Week 40/2009 to Week 39/2023 were analyzed. To account for potential inter‐seasonal variation of sampling intensity, we assessed the sentinel prevalence of influenza A(H3N2); that is, instead of absolute numbers, we used the percentage of A(H3N2) positives relative to the total sentinel samples tested (positivity rate, PR).

**Figure 2:**
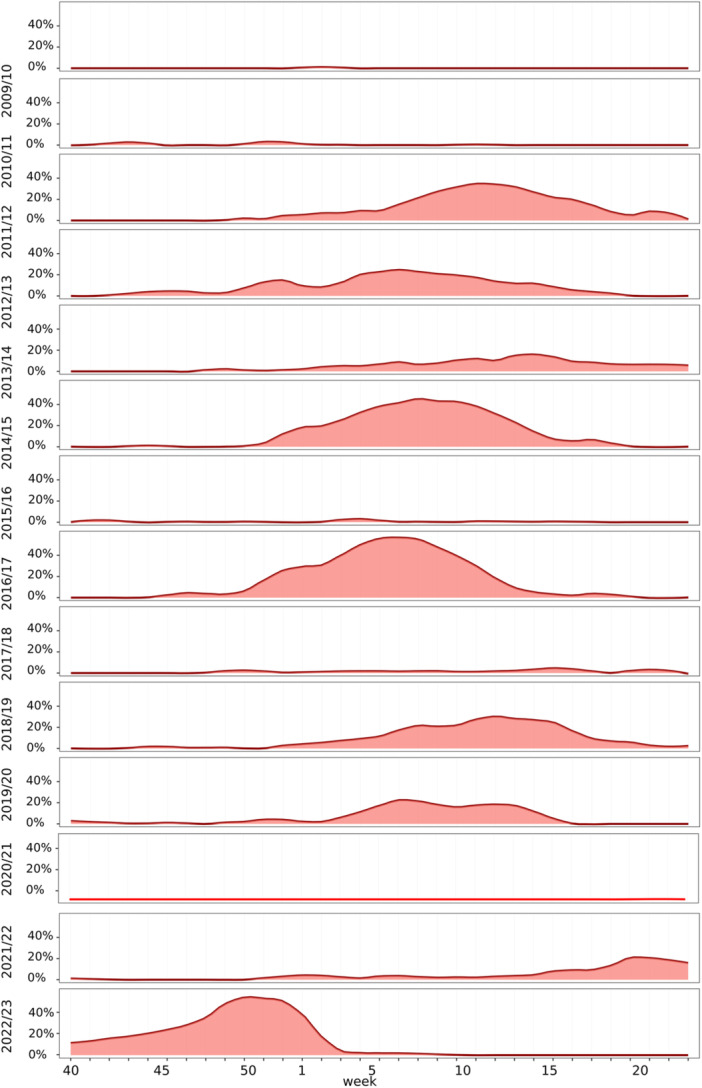
Circulation of influenza A(H3N2) viruses from 2009/10 to 2022/23 in the German ARE sentinel. Shown are the PR of influenza A(H3N2) per calendar week. The reporting period for each influenza season starts in Week 40 and extends through Week 39 of the following year; the *X*‐axis is labeled accordingly.

Both the 2009/10 and 2010/11 season featured extremely low A(H3N2) activity levels with PR ≤ 5%. Afterwards, A(H3N2) influenza viruses circulated at moderate to high levels over four consecutive seasons, from 2011/12 to 2014/15, reaching sentinel PR of 35% (2011/12), 26% (2012/13), 21% (2013/14), and 46% (2014/15) (Figure 2). A subsequent drop in circulation, with positive percentages ≤ 8% throughout 2015/16, was followed by a prominent A(H3N2) wave in 2016/17, when positive percentages exceeded 20% for 11 consecutive weeks. By contrast, the next season (2017/18) was characterized by low A(H3N2) circulation with a positive percentage ≤ 6% throughout. While the 2018/19 season featured a moderate A(H3N2) wave with positive percentages up to 32%, exceeding 20% for a total 8 weeks, the 2019/20 A(H3N2) wave was of lower amplitude and shorter duration (maximum 23%, exceeding 20% for 3 weeks), ending when antipandemic restrictions were introduced. Throughout 2020/21, influenza viruses were practically absent in the sentinel system. Influenza A(H3N2) activity resumed in 2021/22: After a period of sporadic A(H3N2) detections in the 2021 winter, A(H3N2) positive percentages gradually increased beginning Week 10 (March, 2022); Weeks 17–20 met the formal definition of an A(H3N2) influenza wave with positive percentages approaching 20%. This relatively low‐amplitude A(H3N2) influenza wave was significantly delayed compared to the A(H3N2) influenza waves observed during the previous 12 seasons. Continuous summer circulation ensued, reaching levels not observed in any of the previous summers. It was followed by a prominent A(H3N2) fall wave, with positive percentages of approximately 50% during Weeks 48–51/2022. The onset (Week 43/2022), peak (Week 49/2022) and abrupt end (Week 1/2023) of this wave occurred earlier than with any A(H3N2) wave observed during the previous 12 seasons (Figure 2).

### 3.3 Age Groups Affected

**Figure 3:**
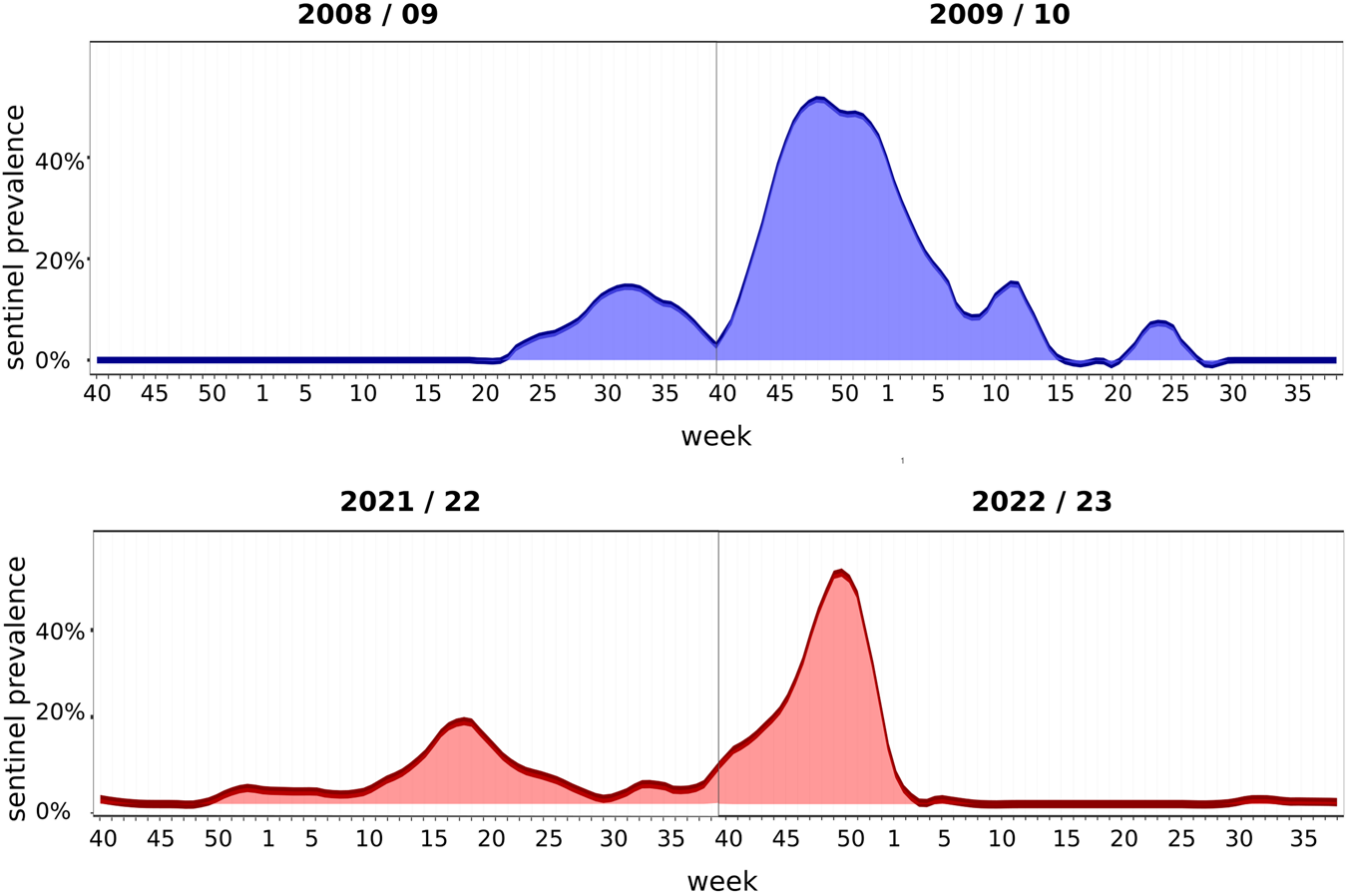
Influenza A(H1N1pdm09) circulation patterns in 2008/09–2009/10 compared with those of influenza A(H3N2) in 2021/22–2022/23. Shown are the percentages of influenza A(H1N1pdm09) and A(H3N2) positive sentinel samples, respectively, per calendar week. The reporting period for each influenza season starts in Week 40 and extends through Week 39 of the following year.

**Figure 4:**
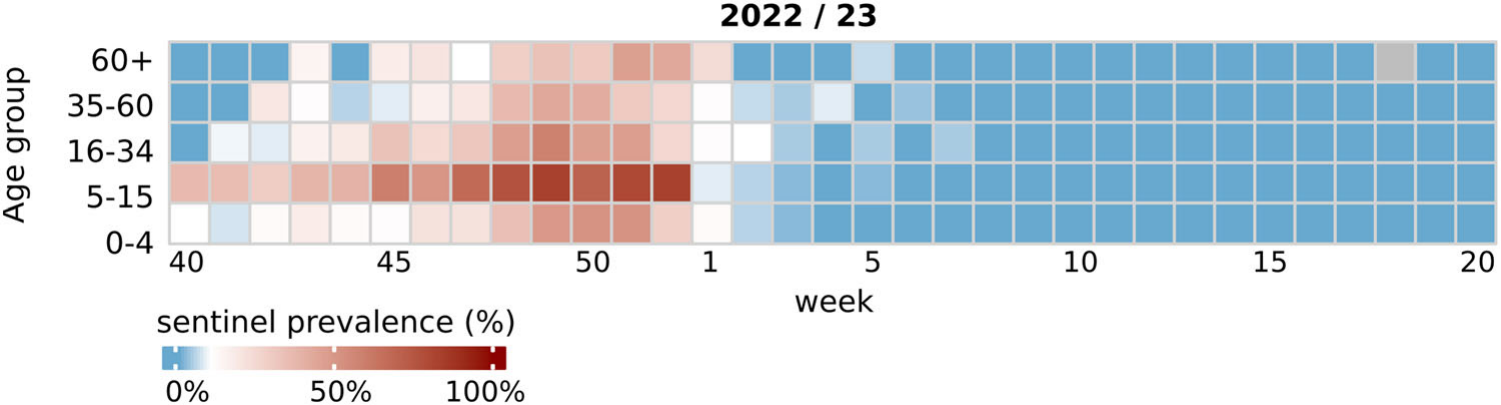
Age distribution of influenza A(H3N2) sentinel cases in the 2022/23 season. Color codes indicate influenza A(H3N2) PR of ARI sentinel patients per age group (*Y*‐axis) and week (*X*‐axis). This figure corresponds to the 12th panel shown in Figure S2, which provides numerical values for each heatmap cell.

**Figure 5:**
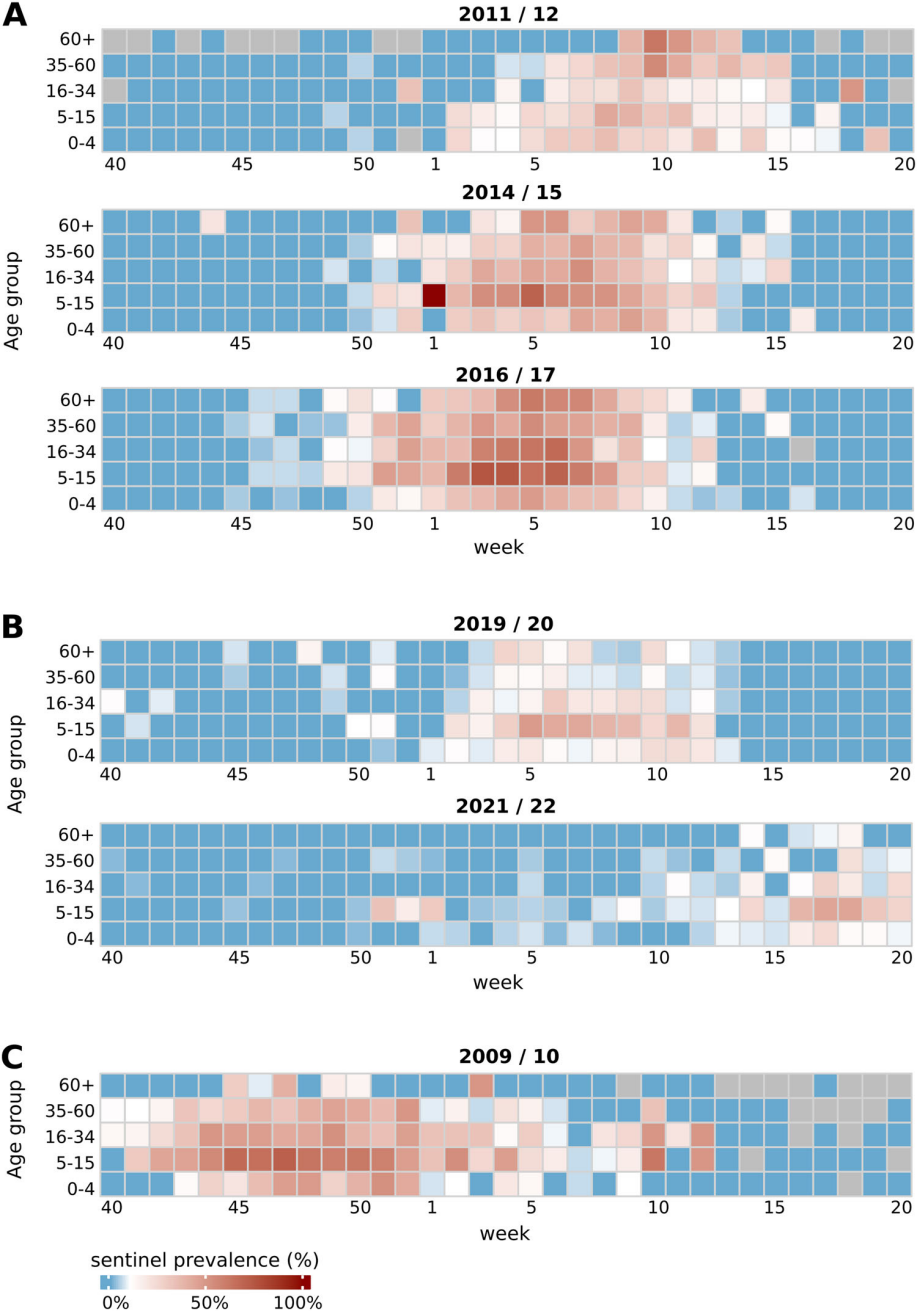
Age distribution of polymerase chain reaction‐confirmed influenza sentinel cases in relevant preceding influenza seasons. (A) A(H3N2), 2011/12, 2014/15, and 2016/17. (B) A(H3N2), 2019/20 influenza season before the COVID‐19 pandemic and A(H3N2), 2021/22 after COVID‐19‐associated restrictions ceased. (C) A(H1N1pdm09), 2009/10. Color codes indicate influenza‐positive percentages of ARI sentinel patients per age group (*Y*‐axis) and week (*X*‐axis). A and B correspond to panels 2, 5, 7, and 10, 12 of Figure S2, respectively.

To better understand the spreading dynamics of the influenza A(H3N2) resurgence, the 2022/23 sentinel prevalences across various age groups were analyzed and compared with those in previous seasons (Figures 2, 3, 4, 5 and S1–S2). The 2022 fall/winter wave began in schoolchildren aged 5–15 years. From Weeks 40 through 52, this age group displayed the highest positivity rates throughout, peaking at 83% in Week 52 (December 2022; Figure 4). Its unusually abrupt end coincided with the German Christmas holidays, which traditionally lead to a significant reduction of contacts among schoolchildren at the turn of the year. The number of samples available during this period is significantly limited. However, because the influenza virus circulation did not continue as in other seasons, this is not a bias. The sudden end of the A(H3N2) wave in school children was accompanied by a sharp decrease of positivity rates in all other age groups (Figure 4). A more granular analysis of sentinel positivity rates, based on narrower age groups, showed that A(H3N2) positive percentages were highest among 10–14‐year old children (Figure S1).

Next, we assessed A(H3N2) sentinel prevalences per age group in selected previous seasons, including the 2011/12, 2014/15 and 2016/17 seasons, which exhibited strong A(H3N2) activity; the 2019/20 season, which preceded the COVID‐19 pandemic; and the 2021/22 season, which saw the first A(H3N2) recurrence after cessation of influenza activity. In each of these seasons, initial increases in A(H3N2) prevalences among 5–15‐year‐olds, and higher peak prevalences compared with that of all other age groups were detected (Figure 5A,B and Figure S2).

Finally, we assessed age group‐dependent influenza A(H1N1)pdm09 prevalences during the 2009/10 influenza A(H1N1) pandemic season. Notably, A(H1N1)pdm09 prevalences showed a similar pattern as with the first influenza wave (A(H3N2) after emergence of COVID‐19, with influenza A(H1N1)pdm09 prevalence rising first, and to the highest levels, in 5–15 year old children (Figure 5c).

Seasonal comparisons show a peak in the number of influenza cases in the first quarter. In periods in which viruses did not circulate for longer, this peak is brought forward to the autumn of the previous year (Figures 2 and 3).

### 3.4 Molecular Analysis of Influenza A(H3N2) Viruses Circulating in 2021/22 and 2022/23

**Figure 6:**
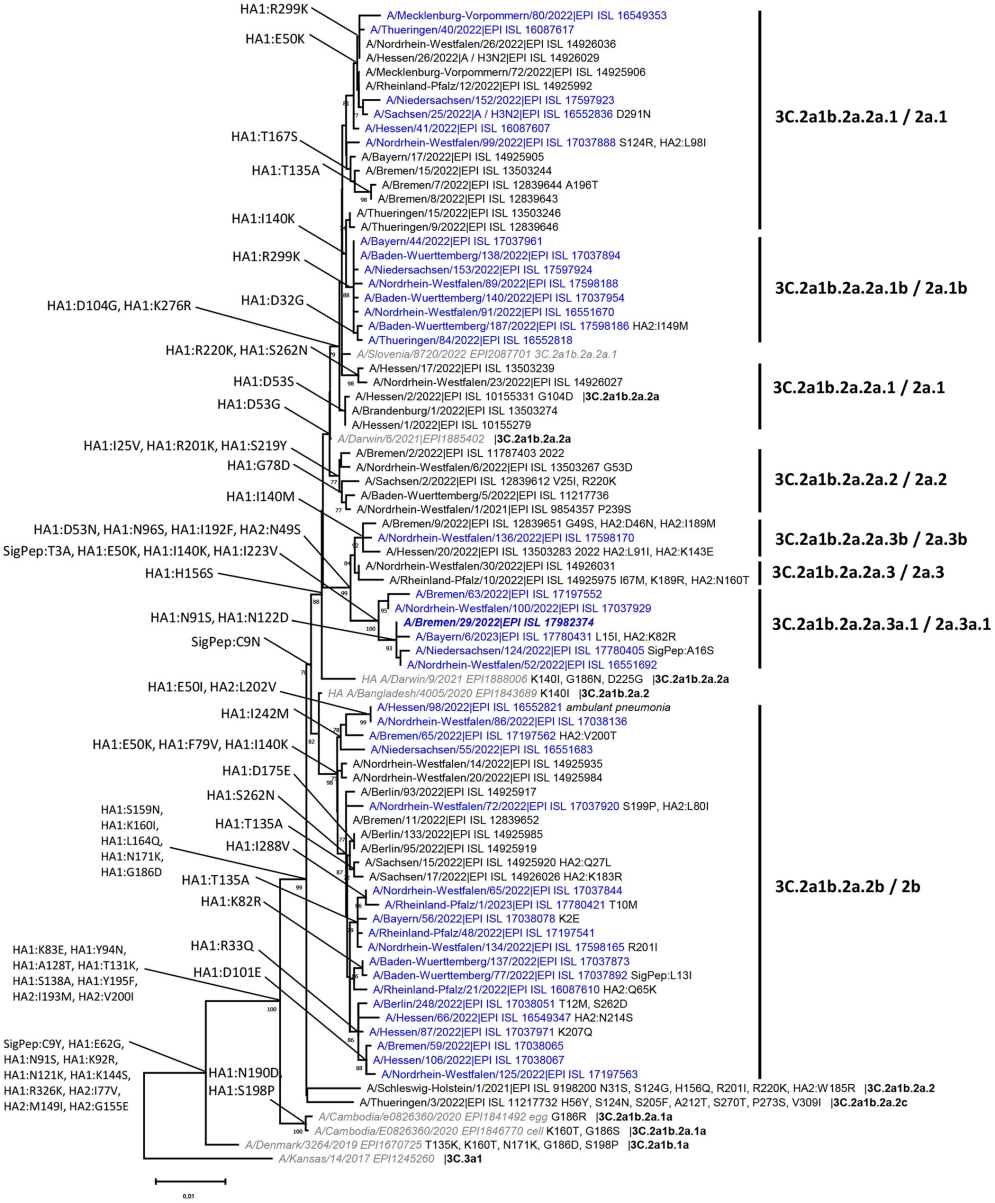
Phylogenetic analysis of the gene coding for hemagglutinin of A(H3N2) influenza viruses circulating in Germany, 2021/22–2022/23. Selected A(H3N2) viruses collected within the German ARI sentinel during the 2021/22 (*n* = 34, black) and 2022/23 (*n* = 40, blue) seasons are shown with ECDC/WHO reference viruses (in italics) and clades according to www.gisaid.org. Clade‐characteristic deduced amino acid substitutions within HA1, HA2, and the signal peptide (SigPep) are displayed at the root of the respective branch. The analysis was performed with Mega (NJ, K2, bootstrap test with 1000 replicates, partial deletion: site coverage cutoff 5%).

The molecular analysis was carried out to see whether certain lines of influenza viruses circulated preferentially after the restrictive measures of the COVID‐19 pandemic. 768 A(H3N2) viruses collected during the 2021/22 and 2022/23 sentinel surveillance underwent WGS. All belonged to clade 3 C.2a1b.2a.2 (short name: clade 2). They were assigned to 10 sub‐clades based on HA‐based clade definitions from GISAID, that formed four distinct clusters in the phylogenetic analysis based on phylogenetic distance: Cluster 1 comprised clades 2a.1(A/Slovenia/8720/2022‐like) and 2a.1b; cluster 2 comprised clades 2a (A/Darwin/9/2021) and 2a.2; cluster 3 comprised clades 2a.3, 2a.3a.1 (A/Bremen/29/2022) and 2a.3b. Additionally, viruses of cluster 4 comprised clade 2b (A/Bangladesh/4005/2020‐like) (Figure 6 and Table S1).

While six HA variants (clades 2, 2a, 2a.2, 2a.3, 2a.3b, 2c) circulated only sporadically in 2022, others were responsible for substantial portions of the influenza A(H3N2) cases at different times of the year. Clade 2a.1 accounted for most sequenced cases during the 2021/22 spring wave, but was responsible for only a minor fraction of these during the 2022/23 A(H3N2) winter wave, which was dominated by three novel virus clades: Clade 2a.3a.1, descending from 2a.3, rose gradually, reaching 8% of sequenced viruses in Weeks 45–47, 2022. Clade 2a.1b, descending from 2a.1, increased to 25% in Week 52, 2022. Clade 2b, which had emerged during the A(H3N2) spring wave, was predominant and associated with 61% of cases in Weeks 41–51, 2022 (Figure S3).

### 3.5 Antigenic Analysis of Influenza Viruses by Hemagglutinin (HA) Inhibition

**Figure 7:**
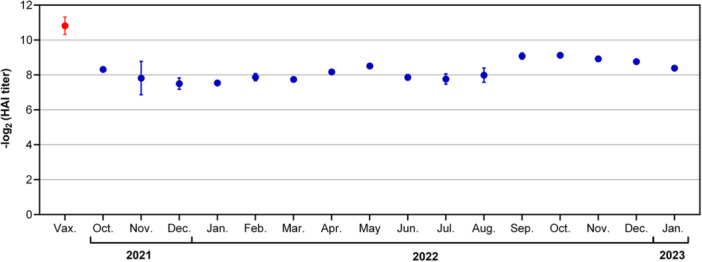
Antigenic analysis of A(H3N2) influenza viruses circulating in Germany 2021/22 and 2022/23. Reactivity of A(H3N2) influenza viruses with antisera raised against the vaccine strains; red circles: reactivity of vaccine viruses; blue circles: reactivity of 699 influenza A(H3N2) viruses isolated in seasons 2021/22 and 2022/23, based on the month of primary sample collection. Shown is for each month the mean ± SEM of the log_2_‐transformed reciprocal titer values.

To evaluate the antigenic correspondence (match) between the A(H3N2) strains circulating in Germany and those included in the vaccine, we analyzed a total 699 A(H3N2) viruses isolated during the 2021/22 (*n* = 350) and 2022/23 (*n* = 349) seasons by HA inhibition assay. All viruses displayed high reactivity with postinfection ferret antisera raised against the Northern hemisphere vaccine strains [A/Cambodia/e0826360/2020 A(H3N2) (2021/22) and A/Darwin/9/2021 A(H3N2) (2022/23), respectively] (Figure 7). Of note, A(H3N2) viruses of the newly emerged clades also reacted well with the specific antisera.

### 3.6 Serological Investigation

**Figure 8:**
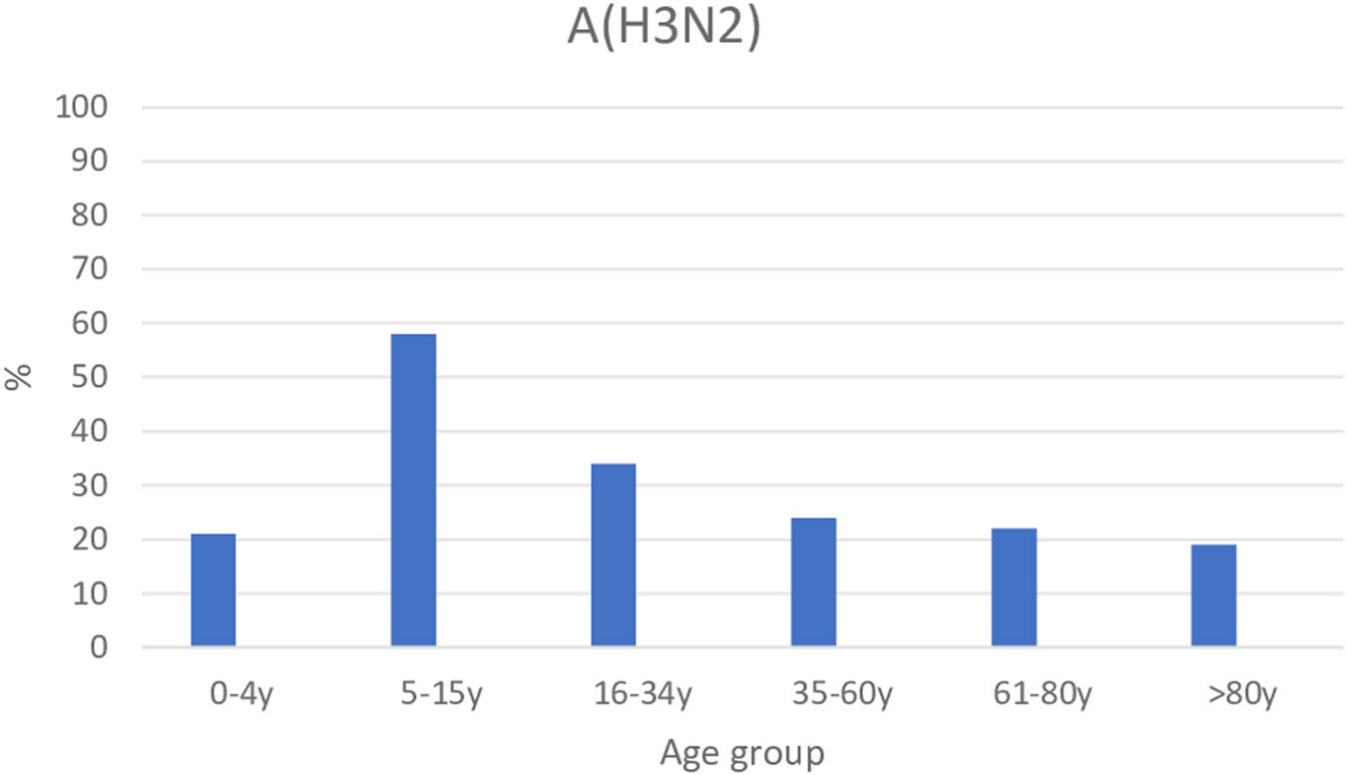
Seroprevalences against A(H3N2) from the move‐in area of Berlin in August 2024.

In unvaccinated individuals, antibody levels 20 months after the end of the A(H3N2) wave still reflected the events of 2022: the 5–15 age group had the highest seroprevalences (Figure 8). Among vaccinees, the highest number was found in those aged > 60 years (Figure S4).

### 3.7 Antiviral Susceptibility of Influenza Viruses to Neuraminidase (NA) Inhibitors, M2 Ion Channel (M2) Inhibitors, and Cap‐Dependent Polymerase (PA) Inhibitors

Altogether, 936 A(H3N2) viruses collected in Germany during the 2021/22 and 2022/23 seasons underwent phenotypic (508), genotypic (794), or both (366) analysis for resistance against NA inhibitors (NAI). NAI resistance, defined as ≥ 10‐fold increase in the IC_50_ of NA inhibitors or presence of NAI resistance‐associated mutations, was not detected in any of the studied viruses.

However, sequence analysis of the M2 gene of 780 viruses demonstrated that the M2‐S31N substitution, associated with resistance to M2 inhibitors was present throughout; for four of these viruses, the molecular resistance marker M2‐L26F was identified additionally. None of the 460 PA sequences had substitutions associated with reduced susceptibility to the PA inhibitor baloxavir marboxil (Table S1).

## 4 Discussion

We present an analysis of influenza A(H3N2) activity in Germany spanning a total of 14 years, based on data from the national laboratory‐based sentinel surveillance for ARIs. During the COVID‐19 prepandemic seasons (2009/10–2019/20), the observed seasonal patterns aligned well with the typical timing of influenza waves in the northern hemisphere [4, 5, 23]. Subsequently, as NPIs were implemented and released during the COVID‐19 pandemic, atypical A(H3N2) circulation patterns were observed. In particular, as the acute phase of the COVID‐19 pandemic in Germany ended and respiratory countermeasures were discontinued, our virological sentinel surveillance registered an A(H3N2)‐driven influenza resurgence that spanned the 2021/22 and 2022/23 seasons and represented a substantial departure from the regular seasonal cycles of A(H3N2) activity in Germany. A similar pattern of two distinct waves, the first at the end of spring (later than the typical influenza spring wave), the second in fall before the turn of the year (earlier than the typical winter wave), with sustained low‐level influenza activity in‐between, had previously only been observed in 2009 when pandemic A(H1N1)pdm09 influenza viruses emerged and spread [24].

Influenza viruses circulate worldwide and exhibit typical seasonal patterns. While peak activity occurs during the winter months in the northern and southern hemispheres, in tropical regions, influenza activity is more closely associated with the rainy season [25]. The link here is the intensity of exposure to light and its effect on the immune system. The sharp decline in influenza activity in spring 2022 in connection with the German Easter holidays shows that the combination of the seasonal factor of light and changes in contact patterns as a result of the holidays led to a rapid decline in influenza activity. Regardless of this, the seasonal factor cannot completely prevent the circulation of viruses in the absence of or with low baseline population immunity.

The atypical temporal patterns of both the 2009 A(H1N1)pdm09 waves and the 2022 A(H3N2) waves are most plausibly explained by deficits in the population's baseline immunity: In 2009, when A(H1N1)pdm09 first entered the human population, these deficits were likely present in all age groups because the virus was antigenically distinct from all previously circulating influenza strains. In 2022, these immunologic deficits were present only in a subset of the population: children who had little chances for repeated exposures to influenza viruses. The likelihood for primary infection had been relatively low for three consecutive winters, given the early end of the 2019/20 wave and the absent or minimal circulation for the following 2 years. Therefore, this immunologically‐naïve subgroup was probably larger than usual in 2022, a notion supported by extraordinarily high A(H3N2) positive percentages in those ≤ 15 years of age. In short, we suggest that unusually high, out‐of‐season infection rates in children were likely due to low baseline immunity in this age group, which triggered the resurgence of A(H3N2) across age groups. Although in 2021 adult immunity still appeared to be protective in the vast majority of individuals, it is possible that the 2022 resurgence was promoted further by a subtle reduction of baseline immunity in the adult population [26].

In 2019/20, both A(H1N1)pdm09 and A(H3N2) influenza viruses circulated. The A(H1N1)pdm09 wave had already passed its peak in early 2020, but COVID‐19 restrictions halted the seasonal A(H3N2) wave. Consequently, fewer individuals were exposed to A(H3N2) than to A(H1N1)pdm09, likely reducing baseline immunity against A(H3N2). This explains plausibly why A(H3N2) viruses resurged first in 2022, earlier and more intensely than A(H1N1)pdm09.

In line with previous seasons of A(H3N2) predominance (e.g., 2014/15, 2016/17), A(H3N2) positive percentages were highest in children aged 5–15 years, especially those aged 10–12 years. This aligns with data from numerous countries indicating that school‐aged children play a key role in the seasonal spread of influenza A due to the dense social interactions in schools [27, 28, 29]. Additionally, robust baseline immunity against influenza viruses may emerge through repeated antigenic exposures, akin to recent observations for SARS‐CoV‐2 [30]. The accumulation of these exposures over time likely enhances the breadth of humoral immunity, leading to increased baseline immunity during infancy post age 15.

The virological diagnostic data is supported by the serological analyses. Even 20 months after the A(H3N2) wave, in September 2024, the age group of 5–15‐year‐olds displayed the highest seroprevalence, at 58%. This value exceeds the seroprevalences observed in other age groups substantially, indicating strongly that other age groups were not infected to the same extent in this wave.

School closures during the Christmas holidays were followed by a drastic reduction in A(H3N2) positivity rates. Similar trends, where school closures due to public holidays resulted in a slight decline (or at least a plateau) of positivity rates among school‐aged children, had been observed in previous seasons (2012/13 and 2016/17); however, the reopening of schools was followed by an influenza resurgence that also spread to other age groups. In contrast, in 2022 the decline in positive percentages post‐Christmas break was not followed by a resurge. The most plausible explanation for this phenomenon is that children, the group with the highest infection rates, had acquired high levels of immunity almost synchronously before the 2022 Christmas break. Consequently, infections were unable to spread after schools reopened. The abrupt cessation of the winter wave affected all age groups, suggesting that children may have played an important role in the spread of this A(H3N2) wave. The suppression of sustained transmission in children, achieved through synchronous acquisition of immunity followed by contact restrictions (due to vacations), appears to have effectively prevented the spread of the virus across different age groups.

Our findings reinforce the notion that children play an important part in the transmission and spread of influenza A virus, leading epidemic waves; and that preventive measures targeting this age group may be particularly effective at reducing the population‐wide spread of influenza virus [27, 29, 31]. High vaccination coverage of school‐aged children benefits to the vaccinated children and the potential indirect protection to families, school staff, and the broader community [27, 28, 32, 33]. The observation that rapid spread of natural infections among 5‐15‐year‐olds, followed by school closures, was associated with a cessation of influenza A activity across all age groups, may inform novel strategies to prevent the spread of the virus: The implication is that near‐synchronous acquisition of high immunity levels among school‐aged children before a school break could potentially halt an influenza wave at population level. To imitate this effect, a coordinated vaccination campaign targeting all school children within a short time span could be considered; precedence exists that rapid, widespread vaccination campaigns are effective in reducing the spread of respiratory viruses [21, 34]. Such a campaign, completed within 2–3 weeks by medical teams visiting schools, might induce population immunity levels among school children similar to those acquired via natural infection in fall/winter 2022. Influenza vaccination has been linked to a three‐quarter reduction in critical illness risk in healthy children and adults [35, 36, 37, 38]. Therefore, high vaccination coverage in school‐aged children is an important public health goal offering direct and indirect community benefits, including to individuals at risk for severe illness [28, 39].

Almost half of the 30 EU Member States currently have a general recommendation for the vaccination of children and/or adolescents, following the recommendations of WHO and other international bodies, who consider vaccination to be the most effective way of preventing influenza directly in children and indirectly in the community in elderly, therefore advising that children should be considered a priority for vaccination even if they are not at risk for severe illness [1, 40, 41].

Germany's 2022 A(H3N2) influenza winter wave was brief, lasting from October to December, but intense. Notably, influenza A(H3N2) viruses co‐circulated with both RSV and SARS‐CoV‐2, straining intensive care capacities across all age groups. The parallel strong circulation of RSV led to a severe burden of respiratory infections in younger children and overwhelmed pediatric hospital wards in Germany. Thus, while non‐pharmaceutical interventions clearly suppress and interrupt viral transmission, their cessation can induce unusual respiratory virus activity patterns, substantially straining healthcare system capacities. In December 2022, Germany experienced excess mortality, with deaths 19% above the 2018–2021 median, reaching 32% above in Week 51 [42]. The influenza epidemic peaked during this period with over 50 000 newly registered cases weekly [43]. In January 2023, nearly 100 000 deaths from all cases were reported to the Federal Statistical Office, a 13% increase compared to January 2019–2022 [44]. The first week of 2023 saw a 26% rise in deaths over previous years, attributed to the seasonal influenza epidemic and a short‐lived increase in SARS‐CoV‐2 infections [42, 44].

Our genetic data demonstrated predominance of three new clades among A(H3N2) influenza viruses circulating in Germany in 2022/23. However, hemagglutinin genetic divergence did not translate to antigenic drift: 349 A(H3N2) viruses isolated in 2022/23 underwent antigenic characterization, displaying efficient reaction with ferret sera against the A(H3N2) vaccine strains, many with < 2 log_2_ difference from the vaccine strain. This suggests a high antigenic match between A(H3N2) vaccine strain and circulating viruses, contrasting with the low to moderate clinical vaccine effectiveness (VE) calculated for the same period [45]. Clinical influenza VE is typically measured using test‐negative study designs, comparing disease risk between vaccinated and unvaccinated groups [46]. However, if vaccine protection is partial (“leaky”) and unvaccinated individuals are infected at higher rates than vaccinated individuals, quickly acquiring natural immunity, the two groups become more similar, causing the VE to appear lower than it is, leading to a downward‐biased result. During periods of pronounced respiratory virus activity, there is increased likelihood of exposure to high infectious doses of virus, leading to breakthrough infections even in vaccinated individuals [47, 48]. Thus, during the 2022/23 A(H3N2) winterwave higher infectious doses may have led to leaky vaccine protection, resulting in an underestimation of clinical VE. In other words, the low clinical VE values do not represent vaccine escape but the impact of high infection rates during a period of intense A(H3N2) circulation.

Although vaccination remains the preferred option for preventing influenza, antiviral therapy is valuable, for example, in cases of vaccine failure [49]. In Germany, as well as internationally, the prevalence of influenza viruses with reduced susceptibility to recommended antivirals remains low.

Taken together, our study used nationwide virological surveillance data to provide a detailed account of the re‐emergence of influenza viruses in Germany after pandemic restrictions ended. Our findings indicate that influenza activity may be atypically timed under conditions of low population baseline immunity, in line with observations in other countries in 2022 [3, 50, 51]. The postpandemic influenza resurgence in Germany was driven by A(H3N2) viruses, the last influenza subtype to circulate before the COVID‐19 pandemic began. These A(H3N2) viruses circulated mainly among 5–15‐year olds and caused two out‐of‐season waves: a minor, delayed spring wave and a major, early fall/winter wave, which subsided abruptly, in temporal association with the winter school break. Our data show that pandemic countermeasures, in this case COVID‐19, can lead to pandemic‐like situations with endemic viruses. Influenza prevention strategies could guide new approaches and should be adapted, reconsidered, and expanded accordingly.

## Author Contributions

Conceptualization: Ralf Dürrwald, Susanne Duwe, and Djin‐Ye Oh. Methodology: Susanne Duwe, Djin‐Ye Oh, Marianne Wedde, Janine Reiche, Barbara Biere. Validation: Susanne Duwe, Djin‐Ye Oh, Marianne Wedde, Janine Reiche, Barbara Biere. Formal analysis: Susanne Duwe, Djin‐Ye Oh, Marianne Wedde, Janine Reiche, Barbara Biere, Thorsten Wolff, Daniela Börnigen, Ralf Ignatius, Max von Kleist, and Ralf Dürrwald. Investigation: Susanne Duwe, Djin‐Ye Oh, Marianne Wedde, Janine Reiche, Barbara Biere, Thorsten Wolff, Ralf Ignatius, and Ralf Dürrwald. Writing – original draft preparation: Susanne Duwe, Djin‐Ye Oh, and Ralf Dürrwald. Writing – review and editing, all authors. Visualization: Daniela Börnigen, Max von Kleist, Djin‐Ye Oh. Supervision: Ralf Dürrwald. Project administration: Ralf Dürrwald. All authors have read and agreed to the published version of the manuscript.

## Ethics Statement

Written informed consent was obtained from all sentinel patients. Ethical approval for the German national surveillance of influenza and other respiratory viruses was granted by the Charité‐Universitätsmedizin Berlin Ethical Board (reference EA2/126/11). Sentinel surveillance is conducted in accordance with German law (§13, §14 of the Protection against Infection Act). All analyses were performed using pseudonymized data.

## Conflicts of Interest

The authors declare no conflicts of interest.

## Permission to Reproduce Material from Other Sources and Clinical Trial Registration

Material from other sources than indicated in chapter “Materials and Methods” were not used. This study characterized the circulating influenza viruses and is not a clinical trial.

## Supporting information


**Supplementary Figure S1:** Age distribution of influenza A(H3N2) sentinel cases in the 2022/23 season, using 5 year‐ age brackets.

**Supplementary Figure S2:** Age distribution of influenza A(H3N2) cases in the national German ARI sentinel during each season, 2010/11 through 2023/24.

**Supplementary Figure S3:** Co‐circulating HA variants of A(H3N2) influenza viruses in 2021/22 and 2022/23 in Germany.

**Supplementary Figure S4:** Vaccination status in % in persons from the move‐in area of Berlin in August 2024 (100 persons per age group were included in the analysis).

TabS1_DuweOh_final.

## Data Availability

The data that support the findings of this study are available on request from the corresponding author. The data are not publicly available due to privacy or ethical restrictions. Data obtained were reported to the ECDC by TESSy (The European Surveillance System). Additionally, all analyzed genome sequences were submitted to GISAID with accession numbers listed in Supplementary Table S1. Due to data protection regulations, individual participant data cannot be made available. With respect to opportunities to collaborate, please contact the authors.
