# Supplementary figures and images for: Atypical Influenza A(H3N2) Activity Patterns in Germany, 2021–2023, and Characterization of Newly Emerged Virus Clades
Source: J Med Virol. 2025 Aug 7;97(8):e70530. doi: 10.1002/jmv.70530 (PMC12330201; doi:10.1002/jmv.70530)

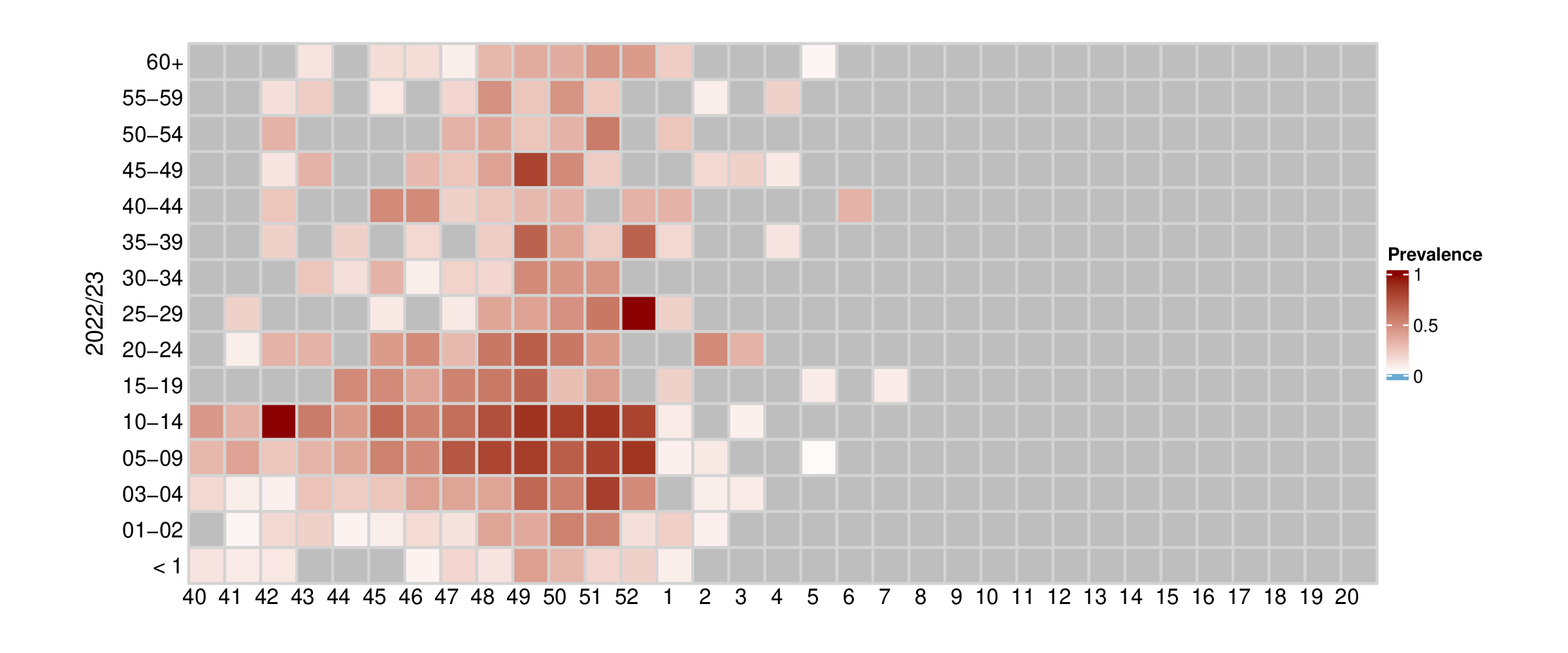

Supplement: Supplementary file 1 — Supplementary Figure S1: Age distribution of influenza A(H3N2) sentinel cases in the 2022/23 season, using 5 year‐ age brackets. [file JMV-97-e70530-s004.png]

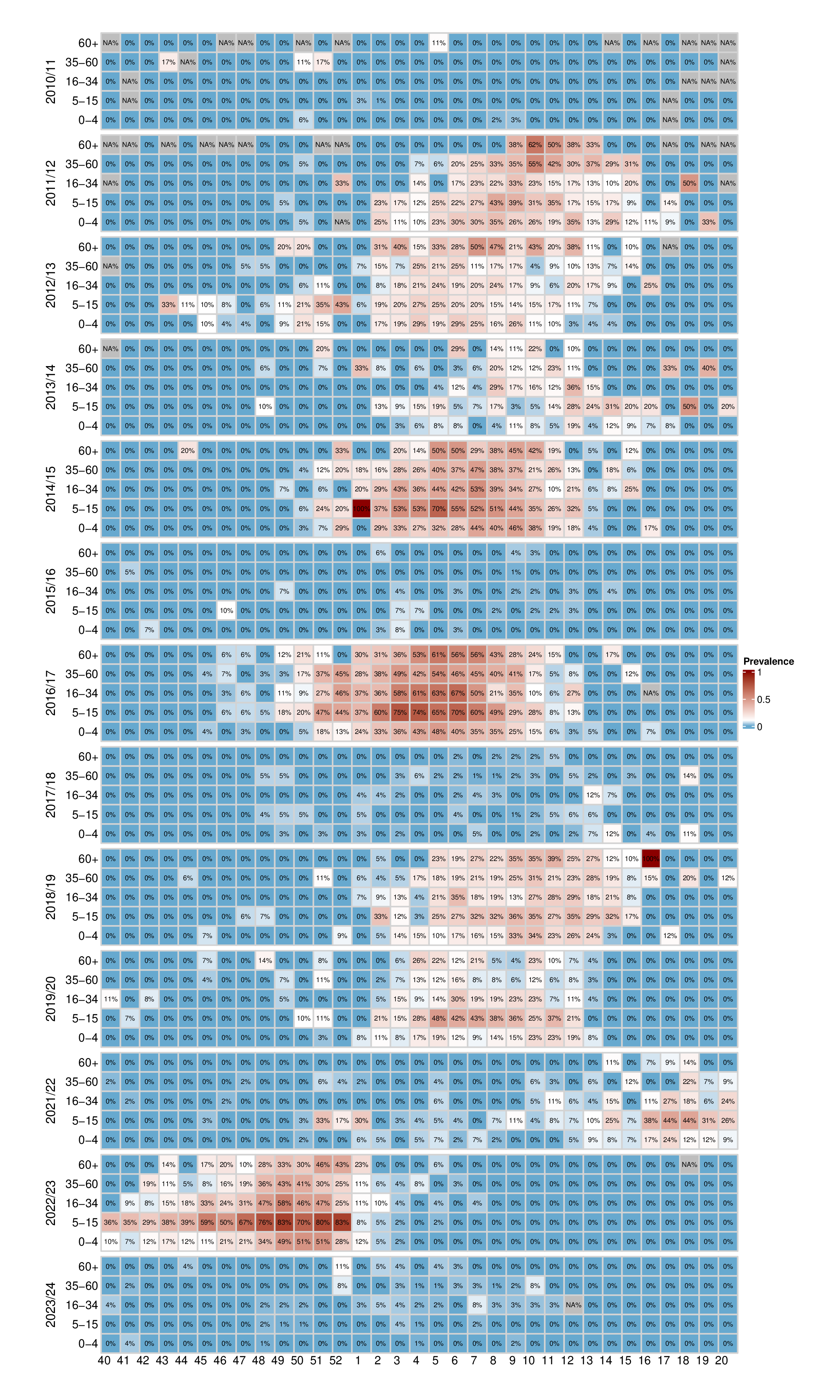

Supplement: Supplementary file 2 — Supplementary Figure S2: Age distribution of influenza A(H3N2) cases in the national German ARI sentinel during each season, 2010/11 through 2023/24. [file JMV-97-e70530-s005.png]

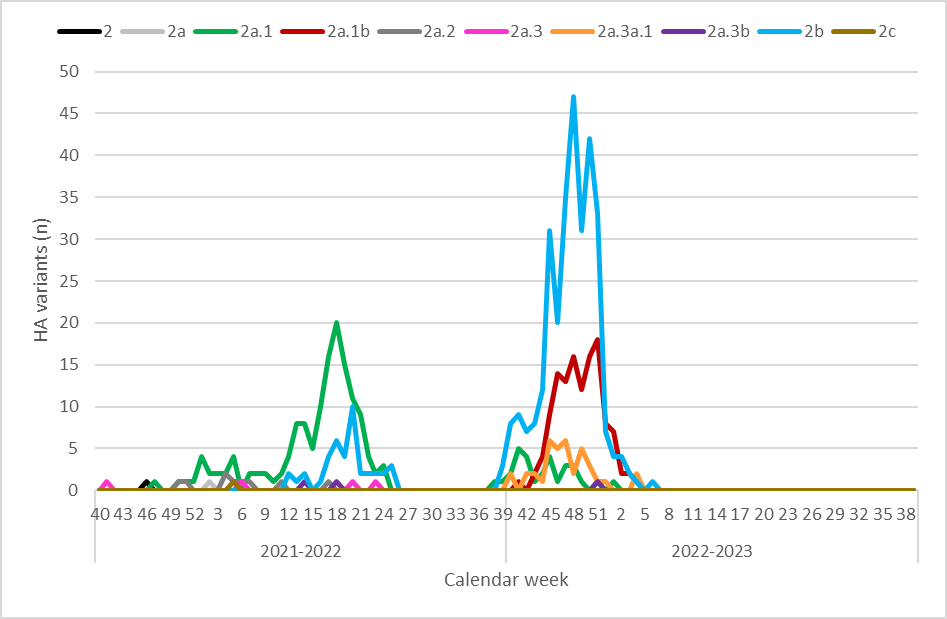

Supplement: Supplementary file 3 — Supplementary Figure S3: Co‐circulating HA variants of A(H3N2) influenza viruses in 2021/22 and 2022/23 in Germany. [file JMV-97-e70530-s001.png]

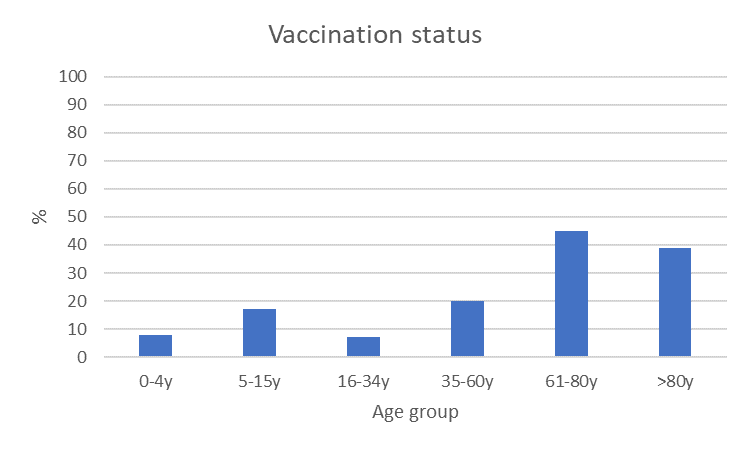

Supplement: Supplementary file 4 — Supplementary Figure S4: Vaccination status in % in persons from the move‐in area of Berlin in August 2024 (100 persons per age group were included in the analysis). [file JMV-97-e70530-s003.tiff]
